# Genetics and Molecular Modeling of New Mutations of Familial Intrahepatic Cholestasis in a Single Italian Center

**DOI:** 10.1371/journal.pone.0145021

**Published:** 2015-12-17

**Authors:** Isabella Giovannoni, Francesco Callea, Emanuele Bellacchio, Giuliano Torre, Jean De Ville De Goyet, Paola Francalanci

**Affiliations:** 1 Dept. Pathology and Molecular Histopathology, Bambino Gesù Children’s Hospital, IRCCS, Rome, Italy; 2 Research Laboratories, Bambino Gesù Children’s Hospital, IRCCS, Rome, Italy; 3 Dept. Hepatology, Gastroenterology and Nutrition Unit, Bambino Gesù Children’s Hospital, IRCCS, Rome, Italy; 4 Dept. Pediatric Surgery and Transplantation, Bambino Gesù Children’s Hospital, IRCCS, Rome, Italy; Texas A&M Health Science Center, UNITED STATES

## Abstract

Familial intrahepatic cholestases (FICs) are a heterogeneous group of autosomal recessive disorders of childhood that disrupt bile formation and present with cholestasis of hepatocellular origin. Three distinct forms are described: FIC1 and FIC2, associated with low/normal GGT level in serum, which are caused by impaired bile salt secretion due to defects in *ATP8B1* encoding the FIC1 protein and defects in *ABCB11* encoding bile salt export pump protein, respectively; FIC3, linked to high GGT level, involves impaired biliary phospholipid secretion due to defects in *ABCB4*, encoding multidrug resistance 3 protein. Different mutations in these genes may cause either a progressive familial intrahepatic cholestasis (PFIC) or a benign recurrent intrahepatic cholestasis (BRIC). For the purposes of the present study we genotyped 27 children with intrahepatic cholestasis, diagnosed on either a clinical or histological basis. Two BRIC, 23 PFIC and 2 BRIC/PFIC were identified. Thirty-four different mutations were found of which 11 were novel. One was a 2Mb deletion (5’UTR- exon 18) in *ATP8B1*. In another case microsatellite analysis of chromosome 2, including *ABCB11*, showed uniparental disomy. Two cases were compound heterozygous for BRIC/PFIC2 mutations. Our results highlight the importance of the pathogenic role of novel mutations in the three genes and unusual modes of their transmission.

## Introduction

Cholestatic disorders are among the most severe liver diseases in infancy and childhood. Cholestasis is defined as an impairment of normal bile flow and is divided into extra-hepatic cholestasis and intra-hepatic cholestasis, the latter can be of hepatocanalicular or ductal origin. Familial intrahepatic cholestases (FICs) are a spectrum of autosomal liver disorders and the two ends are: benign recurrent intrahepatic cholestasis (BRIC) and progressive familial intrahepatic cholestasis (PFIC). The differentiation between BRIC and PFIC is based on phenotypic presentation: BRIC is characterized by intermittent recurrent cholestatic episodes, with irresistible pruritus, mostly without evident liver damage. PFIC is progressive with evolution to end-stage liver disease, occurring in 1/50,000-1/100,000 births [1]. BRICs and PFICs are differentiated as follows: BRIC1 and PFIC1 due to mutations in *ATP8B1* [OMIM 602397], BRIC2 and PFIC2 due to mutations in *ABCB11* [OMIM 603201] and BRIC3 and PFIC3 due to mutations in *ABCB4* [OMIM 171060] [1]. The three types of PFIC have distinctive clinical, biochemical and histological features. PFIC1 or Byler disease [OMIM211600] [2] and PFIC2 or bile salt export pump (BSEP) disease [OMIM601847] [3] are associated with a low or normal serum gamma-glutamyl-transpeptidase (GGT) activity, whereas PFIC3 or multidrug resistance protein 3 (MDR3) disease [OMIM 171060] [4] is associated with a high serum GGT activity.

All three genes encode hepatocanalicular transporters: *ATP8B1* encodes an amino-phospholipid flippase translocating phospholipids from the outer to the inner leaflet of the plasma membrane [5]; *ABCB11* encodes the bile salt export pump, a liver-specific adenosine triphosphate (ATP)-binding cassette transporter [6]; *ABCB4* encodes the multidrug resistance protein 3 functioning as a phospholipid floppase translocating phosphatidylcoline from the inner to the outer leaflet of the membrane [7].

FICs are present worldwide; there is no specific genotype/phenotype correlation and new mutations are continuously reported. The aim of the present study is to report novel mutations and their pathogenetic role and to highlight unusual modes of transmission of FICs observed in a series of 27 patients, thus allowing a correct diagnosis and familial counseling.

## Subjects and Methods

### Patients

We studied 27 children and family relatives who were referred to our center from 2008 to 2015. We have obtained a written informed consent from all patients and parents (when needed) and ethical approval from Bambino Gesù Children's Hospital ethical committee. The clinical diagnosis of FIC was based on the following criteria: (i) hepatocellular cholestasis, (ii) pruritus, (iii) elevated serum bile acid concentrations, (iv) exclusion of other causes of cholestasis. Serological or viral or metabolic markers and histology ruled out other causes of cholestasis of hepatocanalicular (metabolic and neonatal giant cell hepatitis) and ductal (syndromic paucity of biliary duct and ductal plate malformation) origin. Serum GGT activity was used to define the type of FIC.

Thirteen patients (48%) were diagnosed and followed in our center and fourteen cases (52%) were referred to our hospital from other pediatric centers. Three families were consanguineous. One patient had a family history of death due to liver disease. Table 1 summarizes patient and clinical data. Liver transplantation (LT) was performed in 14 patients.

**Table 1:** Patients data.

Patient	Age at clinical diagnosis	Associated Phenotype	Clinical data	GGT (U/L) (5–45)	DB (mg/dL) (0–0.5)	TB (mg/dL) (0–1.5)	Outcome
N.1	20Y	BRIC1	Recurrence of J and P	18 UI/L	4 mg/dl	10 mg/dl	UDCA ^
N.2	23Y	BRIC1	Recurrence of J and P	31 UI/L	7 mg/dl	5 mg/dl	UDCA ^
N.3	10 M	PFIC1	J, P, LF	45 UI/L	5 mg/dl	10 mg/dl	dead
N.4	6 M	PFIC1	J, P, LF	33 UI/L	7 mg/dl	13 mg/dl	OLT
N.5	3 M	PFIC1	J, P, LF	17 UI/L	3 mg/dl	2 mg/dl	OLT
N.6	12M	PFIC1	J, P, LF	12 UI/L	13 mg/dl	5 mg/dl	OLT
N.7	5 M	PFIC1	J, P, LF	20 UI/L	9 mg/dl	5 mg/dl	OLT
N.8	15Y	PFIC2	J, P	12 UI/L	7 mg/dl	11 mg/dl	UDCA ^
N.9	20 M	PFIC2	J, P	26 UI/L	8 mg/dl	11 mg/dl	Bil. Div.
N.10	10 M	PFIC2	J, P, LF	13 UI/L	6 mg/dl	8 mg/dl	OLT
N.11	6 M	PFIC2	J, P, LF	25 UI/L	2 mg/dl	3 mg/dl	OLT
N.12	4Y	PFIC2	J, P, LF	45 UI/L	18 mg/dl	13 mg/dl	OLT
N.13	3 M	PFIC2	J, P, LF	10 UI/L	2 mg/dl	4 mg/dl	OLT
N.14	4Y	PFIC2	J, P	23 UI/L	20 mg/dl	9 mg/dl	§
N.15	3Y	PFIC2	J, P, LF	15 UI/L	3 mg/dl	17 mg/dl	§
N.16	24M	PFIC2	J, P, LF	30 UI/L	23 mg/dl	29 mg/dl	OLT
N.17	20 M	PFIC2	J, P	20 UI/L	2 mg/dl	3 mg/dl	UDCA
N.18	12 M	PFIC2	J, P	43 UI/L	8 mg/dl	5 mg/dl	§
N.19	24M	PFIC2	J, P, LF	27 UI/L	3 mg/dl	3 mg/dl	OLT
N.20	3Y	PFIC2	J, P	40 UI/L	5 mg/dl	9 mg/dl	§
N.21	7M	PFIC2	J, P, LF	37 UI/L	1 mg/dl	4 mg/dl	OLT
N.22	3Y	PFIC2	J, P, LF	25 UI/L	19 mg/dl	10 mg/dl	OLT
N.23	2 M	PFIC2	J, P	32 UI/L	1 mg/dl	9 mg/dl	§
N.24	6M	PFIC2	J, P	18 UI/L	20 mg/dl	13 mg/dl	§
N.25	5Y	PFIC3	J, P, LF	337 UI/L	3 mg/dl	6 mg/dl	OLT
N.26	19M	PFIC3	J, P, LF	656 UI/L	10 mg/dl	7 mg/dl	OLT
N.27	19Y	PFIC3	J, P	500 UI/L	1 mg/dl	4 mg/dl	§

J: jaundice; P: pruritus; LF: liver failure; TB: Total bilirubin; DB: Direct bilirubin; Bil. Div.: Biliary Diversion

^ UDCA during pruritus/ jaundice episodes

§: we have seen the patient only for molecular analysis.

In three families with a patient either deceased or severely affected by PFIC, types 1 and 2, prenatal molecular diagnosis was performed.

All patients were treated with Ursodeoxycholic acid (UDCA; 200mg/die x 2).

Liver Histology and Immunohistochemistry

Thirteen liver biopsies and nine explanted livers were analyzed using formalin-fixed and paraffin embedded tissue; 3 μm-thick sections were stained with hematoxylin and eosin (HE), period acid-Schiff (PAS), period acid-Schiff after diastase digestion (PASD) and Masson trichrome.

Immunohistochemistry (IHC) was done with anti-BSEP (goat polyclonal Ab, 1:100 working dilution, Santa Cruz Biotechnology), anti-MDR3 (mouse monoclonal Ab, 1:50 working dilution, Millipore, clone P3 II-26) and anti-cytokeratin 7 (CK7) (pre-diluted, NeoMarkers antibody) antibody as previously described [8, 9].

### Genomic DNA Sequence Analysis and Real-Time Quantitative PCR

DNA of the 27 patients and their parents was extracted from blood (QIAmp DNA Mini kit, Qiagen). Mutation screening was done using polymerase chain reaction (PCR) amplification and DNA sequencing of coding exons and all splice junctions of *ATP8B1* (NM_005603.4), *ABCB11* (NM_003742.2) and *ABCB4* (NM_000443.3) (S1 Table). Mendelian inheritance was demonstrated by sequencing affected exons in both parents; 300 control alleles were used to analyze new non-synonymous variants and to exclude single-nucleotide polymorphisms. Real-time quantitative PCR (qPCR) experiments were performed to study gross deletion or duplication (S1 Table) using SYBR Green chemistry on ABI PRISM® 7500 (Applied Biosystems). The beta-2-microglobulin gene (B2M,NM_004048) was used as reference gene.

Prenatal diagnosis was performed on genomic DNA isolated from chorionic villi, obtained between 12 and 16 weeks of pregnancy. Maternal DNA contamination was excluded by short tandem repeat typing at 15 loci distributed throughout the human genome, using the PowerPlex16 system.

### Homology Modeling

Homology modeling of BSEP structure was made using the crystal structure of mouse multidrug resistance protein 1A (MDR1A) (Protein Data Bank, PDB, entry 4M1M, chain A) as the template, as the two protein share 50% amino acid identity and belong to the TC3.A.1.201 subfamily of ABC transporter proteins. Modeling of BSEP in the amino acid region 45–1318 was carried out as follows: starting from the backbone atoms of the template, the residues were renamed to as in BSEP according to the pairwise sequence alignment in S1 Fig, and the side chains were generated with ModRefiner [10]. Final model refinement was carried out applying maximum restraints for closer similarity with the template. BSEP loops (residues 102–120 and 659–728) were not modeled.

Homology modeling of the structure of human ABCB4 protein (in the amino acid interval 40–1281) was obtained employing the same procedure as explained above. The crystal structure of mouse MDR1A (PDB entry 3G5U, chain A) sharing 75% amino acid identity with human ABCB4, was used as the template according to the pairwise sequence alignment in S2 Fig (loops were not modeled).

## Results

### IHC and Molecular Results

Patients were classified according to immunohistochemical and molecular results (Table 2). Genomic DNA sequence analysis for FIC-associated genes identified 27 patients; 11 new mutations amongst the 34 found (32%). Seven of the patients had mutations in *ATP8B1*, 17 in *ABCB11* and 3 in *ABCB4*, representing 26%, 63% and 11% respectively. Fourteen patients (52%) were transplanted, one was treated with partial external biliary diversion and one died from PFIC-related end-stage liver failure.

**Table 2:** FIC mutations and their associated phenotype.

Patient	Gene	Allele 1		Allele 2		IHC anti-BSEP	IHC anti-MDR3	References
		Nucleotide change	Amino acid change	Nucleotide change	Amino acid change			
N.1	*ATP8B1*	c.3069_3070delAA	p.Q1023fsX	Not Found		+	+	[11]
N.2	*ATP8B1*	c.208G>A	p.D70N*	c.886C>T	p.R296C	+	+	[11]; [12]
N.3	*ATP8B1*	c.2788C>T	p.R930X	c.2788C>T	p.R930X	+	+	[11]
N.4	*ATP8B1*	c.2097+2T>C	-	c.3040C>T	p.R1014X	na	na	[5]; [11]
N.5	*ATP8B1*	**c.3284G>A**	**p.W1095X**	**c.3284G>A**	**p.W1095X**	+	+	
N.6	*ATP8B1*	c.1336G>A	p.G446R	c.1336G>A	p.G446R	+	+	[13]
N.7	*ATP8B1*	**c.del958_967fsX14**	**p.M320VfsX13**	**del5'UTR-ex18**	-	na	na	
N.8	*ABCB11*	c.403G>A*	p.E135K*	**c.3297delC**	**p.L1099LfsX38**	±	+	[14]
N.9	*ABCB11*	c.3458G>A	p.R1153H	c.3148C>T*	p.R1050C*	+	+	[15]
N.10	*ABCB11*	c.2494C>T	p.R832C	c.2494C>T	p.R832C	-	+	[15]
N.11	*ABCB11*	**c.2005A>G**	**p.I669V**	c.1708G>A	p.A570T	-	+	[16]
N.12	*ABCB11*	c.1621A>C	p.I541L	c.1621A>C	p.I541L	-	+	[17]
N.13	*ABCB11*	c.1621A>C	p.I541L	c.1621A>C	p.I541L	-	+	[17]
N.14	*ABCB11*	**c.1873insA**	**p.T625NfsX5**	c.1708G>A	p.A570T	na	na	[16]
N.15	*ABCB11*	**c.1709C>T**	**p.A570V**	**c.1709C>T**	**p.A570V**	-	+	
N.16	*ABCB11*	**c.1822_1823insCA**	**p.H609HfsX46**	**c.1822_1823insCA**	**p.H609HfsX46**	-	+	
N.17	*ABCB11*	c.154C>T	p.R52W	c.1844A>G	p.H615R	±	+	[8]
N.18	*ABCB11*	c.2787_2788insGAGAT	p.K930EfsX49	c.3457C>T	p.R1153C	-	+	[18]; [6]
N.19	*ABCB11*	c.2842C>T	p.R948C	**c.3081T>A**	**p.S1027R**	na	na	[15]
N.20	*ABCB11*	c.2842C>T	p.R948C	**c.3081T>A**	**p.S1027R**	na	na	[15]
N.21	*ABCB11*	c.1445A>G	p.D482G	c.1445A>G	p.D482G	-	+	[6]
N.22	*ABCB11*	c.1409G>A	p.R470Q	c.1409G>A	p.R470Q	na	na	[19]
N.23	*ABCB11*	c.1409G>A	p.R470Q	c.1409G>A	p.R470Q	na	na	[19]
N.24	*ABCB11*	**c.912T>G**	**p.Y304X**	**c.912T>G**	**p.Y304X**	na	na	
N.25	*ABCB4*	c.1783C>T	p.R595X	c.1783C>T	p.R595X	+	-	[9]
N.26	*ABCB4*	c.1783C>T	p.R595X	c.937_992ins/del6	-	+	-	[9]
N.27	*ABCB4*	**c.1442T>G**	**p.L481R**	c.1954A>G	p.R652G	+	-	[20]

Novel mutations described in this study are shown in bold; BRIC mutations are indicated with *

na: not available.

#### FIC1 cases

FIC1 mutations were identified in 7 (26%) of the 27 patients. Sequence analysis revealed 10 different mutations; three were novel (Table 2).

Case 3, homozygous for the nonsense mutation p.R930X, died due to end-stage liver failure while waiting for LT. For the three subsequent pregnancies prenatal molecular diagnosis were done.

Case 4 presented severe liver failure and underwent liver transplantation. A prenatal molecular diagnosis was provided for the next pregnancy.

In case 7 sequence analysis revealed a homozygosity for p.M320VfsX13; segregation in parental DNA showed the same mutation in the father and wild type condition in the mother. To verify the presence of a deletion we performed a qPCR that revealed a 2Mb deletion (5’UTR- exon 18) in the mother and in the proband.

#### FIC2 cases

FIC2 mutations were identified in 17 (63%) of the 27 patients. Sequence analysis revealed 20 different mutations 7 of which were novel (Table 2). IHC with anti-BSEP showed absence of canalicular label in all cases out 3, while MDR3 expression was preserved.

**Fig 1:**
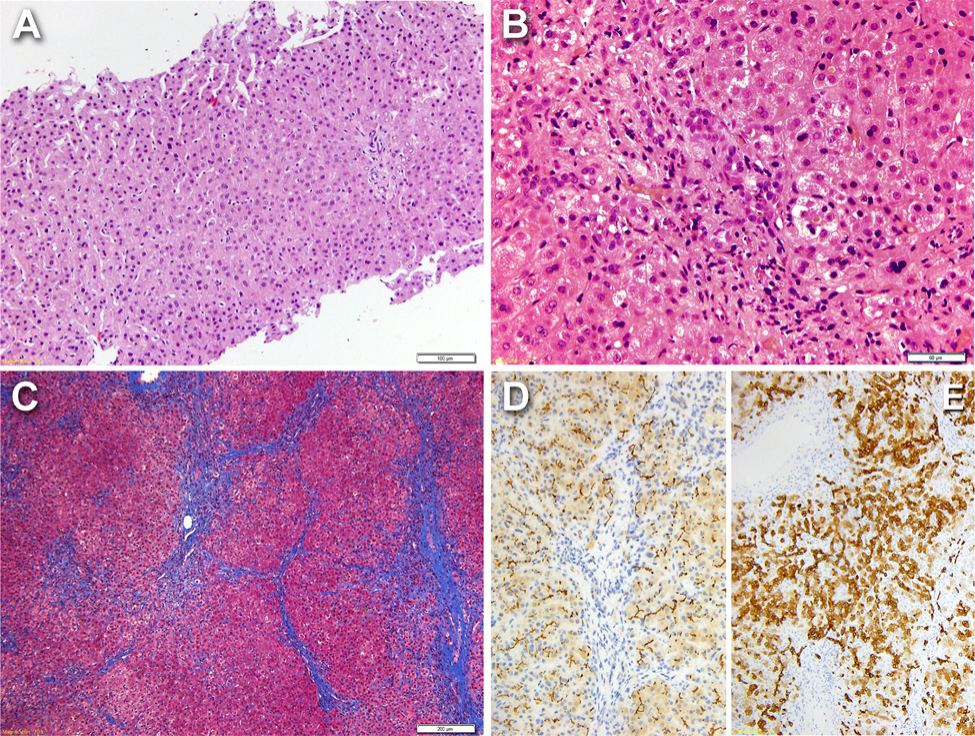
(A) Liver biopsy in case 8 at 11 years of age shows preserved architecture, (HE; 10x). Liver biopsy in case 9 at 6 years of age shows (B) mild intralobular intrahepatic cholestasis (HE, 40x) and (C) fibrosis (Masson trichrome 10x); (D) IHC with anti-BSEP shows strong and diffuse staining at the canalicular edge as in control liver (not shown) (20x); (E) IHC with anti-cytokeratin 7 highlights reactive bile ductules and phenotypically modulated hepatocytes (10x).

Patient 8 presented intermittent jaundice since the age of 1 month and liver biopsies at 11 and 16 years showed normal liver architecture and mild signs of intrahepatic cholestasis (Fig 1A). IHC with anti-BSEP showed mild and focal label at the canalicular membrane, in contrast to the control liver (data not shown). Genetic analysis identified a compound heterozygosity: p.E135K (BRIC mutation) and p.L1099LfsX38 (new PFIC mutation).

Case 9 suffered from intractable pruritus and jaundice since 5 months of age and liver biopsies obtained at 1, 2, 5 and 6 years displayed a progression of cholestasis and bridging fibrosis (Fig 1B, 1C and 1E). IHC showed normal anti-BSEP canalicular staining (Fig 1D). Genotyping showed the p.R1050C BRIC and p.R1153H PFIC mutations. At the age of 6 years, the patient was treated with partial external biliary diversion (cholecystojejunostomy) allowing discharge of bile and disappearance of the pruritus.

In case 10, sequence analysis revealed homozygosity for p.R832C; segregation in parental DNA showed the presence of uniparental disomy in the child [21].

Case 11 was a compound heterozygote for p.A570T and p.I669V, the latter being a novel mutation, and underwent LT. A prenatal molecular diagnosis was done for the next pregnancy.

Case 16, homozygous for the novel mutation p.H609HfsX46, underwent liver transplantation. In the explanted cirrhotic liver a 3cm nodule was identified. On histology the nodule corresponded to a well-differentiated hepatocellular carcinoma (HCC). IHC with anti-BSEP did not show staining either in the cirrhotic liver or in the HCC (data not shown).

In case 17 DNA sequencing revealed two novel mutations. Segregation analysis, carried out in her parents, revealed two mutations, one inherited from the mother (p.H615R) and the other being a *de novo* [8].

#### FIC3 cases

FIC3 mutations were identified in three (11%) of the 27 patients (Table 2). Sequence analysis revealed four different mutations one of which were new. IHC with anti-MDR3 showed absence of canalicular label while BSEP expression was normal. Two patients underwent to LT

### Prediction of Functional Consequences of Variants

The three new mutations in *ATP8B1* (del5'UTR-ex18, p.M320VfsX13 and p.W1095X) led to the total or partial loss of the protein.

**Fig 2:**
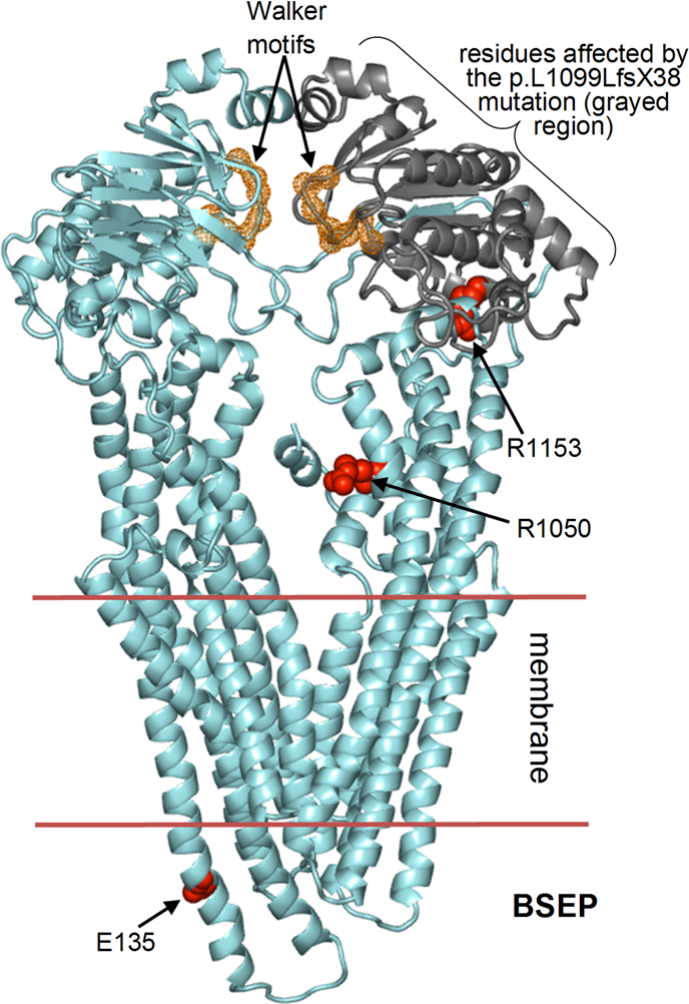
Homology model of BSEP. The amino acids affected by the E135K, R1050C, and R1153H missense mutations are represented by the red spheres. The residues affected by the L1099LfsX38 mutation are indicated by the grayed protein backbone. The two Walker motifs are highlighted with the orange backbone spheres. The transmembrane region is comprised between the two horizontal segments.

The p.E135K mutation has been reported to produce mature BSEP with some reduction in the protein levels [14]. According to the homology model (Fig 2) Glu 135 is located in a helical extracellular region with the side chain fully solvent exposed. Despite the E135K amino acid change produces a reversal of electrostatic charge, it maintains similar helix formation propensity and should not produce important structural changes. However, the E135K mutation might influence the N-glycosylation of the nearby asparagines 109, 116, 122, and 125 that have been reported to undergo this post-translational modification [22]. This could explain the slight reduction observed in the BSEP levels, as proper glycosylation patterns are required for the stability, intracellular trafficking, and function of the protein in the apical membrane [22].

The p.A570V mutation in ABCB11 introduces steric hindrance that interferes with the packing of three helices formed by residues 512–519, 525–534, and 561–575 in the first ABC transporter domain; p.S1027R replaces a serine with an arginine residue in a transmembrane helix, influencing electrostatic properties and the packing of this helix with adjacent transmembrane helices. p.H609HfsX46 and p.T625NfsX5 mutations produce loss of the C-terminal part of the first ABC transporter domain, the second ABC transmembrane domain, and the second ABC transporter domain. The mutation p.K930EfsX49 causes loss of an important part of the last 2 transmembrane domains and the loss of the second ABC transporter domain.

Analysis of the effects of p.R1050C mutation revealed an important reduction in BSEP levels and activity [23, 24].

The p.L1099LfsX38 mutation causes loss of most of the residues forming the second ABC transporter domain (Fig 2), thereby abolishing the protein function.

The c.3458G>A (p.R1153H) mutation of *ABCB11* produces severe splicing alteration resulting in only 3% of wild-type-like splicing product [14]. In a patient carrying the homozygote p.R1153H mutation BSEP protein was not detected [15].

Homology modeling of ABCB4 suggests that the single new mutation p.L481R disrupts a hydrophobic pocket that destabilizes the first ABC transporter domain (S1 Model).

## Discussion

Familial intrahepatic cholestases (FICs) refer to a heterogeneous group of autosomal recessive liver disorders, which occur worldwide. Molecular analysis is mandatory for a correct and complete diagnosis. In all cases of FIC, a wide range of variations in clinical phenotypes has been observed [11–13, 15, 18–20, 25], possibly due to variability in mutations in all the genes and in the type of segregation, more frequently compound heterozygosity than homozygosity.

The 27 patients presented clinical signs consistent with FICs. Genetic analysis for *ATP8B1*, *ABCB11* and *ABCB4* was carried out in all the patients. Of the 34 mutations detected, 11 (32%) were novel (Table 2).

The 11 new mutations detected in our series fall into four categories: 1 gross deletion, 2 nonsense, 4 missense and 4 frameshift mutations. Missense mutations were the most frequent variations found in our cohort (20/34; 59%). The effect of missense mutations is difficult to predict, unlike nonsense and frameshift mutations, which are easily predictable as being destructive. Segregation analysis, study of 300 alleles and homology modeling helped to distinguish polymorphisms from pathogenic variants. Segregation studies were performed in 21 of the 27 families; 89% of the patients showed a normal mendelian inheritance. In three families the probands (cases 7, 10 and 17) showed a clinical and histologic diagnosis of PFIC and a homozygous or compound heterozygous genetic condition, but only one parent displayed a mutation. In these three families additional specific molecular analyses were performed. In case 7, a qPCR for *ATP8B1* showed the presence of a 2Mb deletion, including 5’UTR to exon 18, in both the mother and proband. This gross deletion is new and is so far the largest deletion described in FIC1. Gross deletions like this are relatively rare in all three forms of FICs and are therefore difficult to identify. In case 10, microsatellite analysis revealed a paternal segmental isodisomy in a homozygous proband with a heterozygous father and a wild-type mother. Segmental isodisomy is a form of uniparental disomy where a given subject inherits 2 copies of a chromosome from one parent and no copy from the other, as previously reported [21]. In case 17, homology modeling, carried out to study two novel mutations (p.R52W and p.H615R) and segregation analysis, performed in both parents, highlighted the pathogenetic role and revealed that one mutation was inherited from the mother (p.H615R) and that the other was a *de novo* mutation, as mutations or a potential mosaicism were ruled out in the natural father [8].

In our cohort only case 16 has developed HCC; it is difficult to draw conclusions about the oncopathogenetic role of p.H609HfsX46 mutation: whether the development of HCC is due to the mutation, although it is a frameshift mutation in homozygous, or to the condition of cirrhosis.

**Fig 3:**
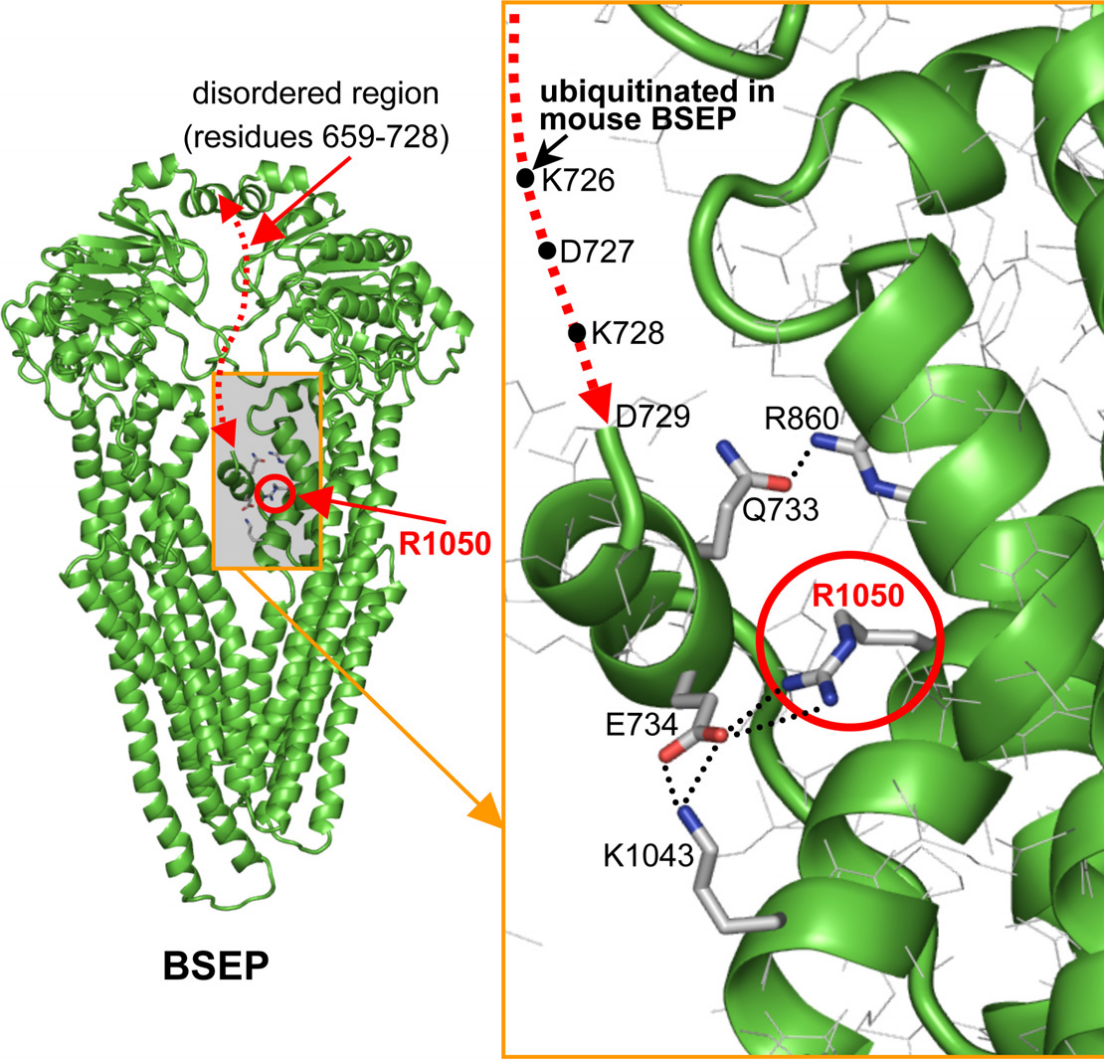
Model of BSEP and detailed view around the site of the R1050C mutation. Shown as sticks are the residues that stabilize, via non-covalent interactions, the short helix contiguous to the 659–728 disordered region (red dotted line). Hydrogen bonds and salt-bridges are indicated by black dots between residues. The schematic positions of the three most C-terminal residues of the disordered peptide are indicated. These include Lys 726, the residue found to be ubiquitinated in the mouse BSEP.

Progression from a benign to a severe form has rarely been reported in the literature [26]. This is an important point and seems to apply to two our cases. Indeed, in our cohort there were two patients with BRIC and PFIC mutations, presenting an intermediate phenotype. In case 8, genetic analysis showed a compound heterozygosity p.E135K, p.L1099LfsX38. The p.E135K may have an effect on glycosylation, consistent with a trafficking modification without the total loss of protein function. Studies on the effects of various BSEP mutations have determined that p.R1050C reduces the expression level (by 30% to 50% compared to wild-type) and reduces transport activity by up to 50% [23] but does not significantly impair the processing of this protein [24]. With regard to the latter phenomenon, some insights may be gained by inspecting the homology model of BSEP protein. It can be seen that Arg 1050 (site of the R1050C mutation) is engaged in a salt bridge with Glu 734, which helps to anchor the short helix formed by residues 731–735 to the remainder of the protein structure (Fig 3). In the mouse homologue of BSEP, Lys 727 (corresponding to Lys 726 in human BSEP) has been found to undergo ubiquitination [27]. This lysine is located at the extremity of the disordered 659–728 amino acid region that is directly connected to the Glu 734-containing helix. It can therefore be envisaged that the R1050C replacement abrogates the salt bridge with Glu 734, thus weakening the anchorage of the short 731–735 helix increasing its mobility together with adjacent residues including Lys 726 (Fig 3). This lysine should undergo ubiquitination and, like in the mouse homologue, the R1050C mutation will have the functional consequence of the enhancement of Lys 726 exposure to ubiquitinating enzymes. The p.R1153H (c.3458G>A) produces severe splicing alteration leading to only 3% of wild-type-like splicing product [14], consistent with the lack of detectable BSEP protein in a patient carrying the homozygote mutation [15]. The eventual tiny residual fraction of correctly spliced BSEP protein is not expected to be capable of proper transport activity, since the p.R1153H mutation affects residues interacting with and determining the structure of the Walker motif in the second ABC transporter domain of BSEP (Fig 2). However, p.R1153H appears to be more severe than p.R1050C, with destabilizing effects (see homology modeling results). Previous subcellular distribution studies have revealed that the R1050C mutant is mainly located along the canalicular membrane, whereas the other PFIC2 mutants remain intracellular; also in that study, the transport activity was found to be 50–60% in BRIC2 mutants and 0–30% in PFIC2 mutants compared to wild-type [23]. Furthermore, in case 9, BSEP expression was preserved when compared to healthy control liver. These observations appear to support the possibility that the less severe mutation (R1050C) may compensate the more severe R1153H, but not sufficiently to maintain the minimum level of transport activity expected in BRIC condition. The phenotype would be intermediate between BRIC and PFIC, but more severe than that observed in case 8. Partial external biliary diversion in case 9 reduced pruritus. Our experience points to the existence of phenotypes with different degrees of severity: patients carrying BRIC/PFIC mutations might present a spectrum of phenotypes depending on the severity of the mutations.

## Conclusion

The present study has confirmed that IHC for BSEP and MDR3 deficiency is an useful and cheap method in case of suspected FIC. Indeed a reduction in protein expression at the canalicular membrane is found in more than 90% of FIC2 patients [15]. However, a canalicular membrane label cannot rule out a deficiency condition. Therefore, when immunostaining for BSEP and MDR3 is detectable, but clinical signs evoke a strong suspicion of FICs, sequencing analysis is recommended. When structural data of related proteins are available, homology modeling can usefully assist in the investigation of the effects of novel mutations. The outcome of patients with PFIC is usually poor due to the natural history and complications (biliary cirrhosis, untreatable pruritus, extrahepatic manifestations) and sometimes, as demonstrated in this study, early (childhood-adolescence) liver transplantation may be required. Moreover, we have confirmed that prenatal diagnosis of PFIC1-3 is feasible and that this information is useful for genetic counseling and subsequent pregnancies in families with affected children.

Other findings of the study, such as novel mutations and unusual mode of transmission, together with the rarity and complexity of these diseases, suggest the opportunity of centralizing patients in a single reference centre with the necessary expertise in the field of genetic liver disease.

## Supporting Information

S1 Fig Pairwise sequence alignment (clustal format) of human BSEP and multidrug resistance protein 1A (MDR1A) from mouse. The grayed residues in MDR1A sequence correspond to a disordered region with atomic coordinates missing in the crystal structure employed as the template for homology modeling (PDB accession code 4M1M, chain A). The two grayed regions in BSEP sequence highlight, in the order, an insertion with respect to MDR1A and to a region homologous to the disordered region of MDR1A. Amino acids highlighted in red indicate the residues affected by the mutations E135K, R1050C, R1153H, and the position of the first residue involved in the L1099LfsX38.
(DOC)

S2 Fig Pairwise sequence alignment of human ABCB4 and multidrug resistance protein 1A (MDR1A) from mouse. The grayed residues in MDR1A sequence correspond to a disordered region with atomic coordinates missing in the crystal structure employed as the template for homology modeling (PDB entry 3G5U, chain A).
(DOC)

S1 Model Atomic coordinates (PDB format) of the homology model of human MDR3. (PDB)

S1 Table Primers for PCR and qPCR amplification of *ATP8B1*, *ABCB11* and *ABCB4* genomic DNA. (DOCX)
