# Supplementary material for: Genetics and Molecular Modeling of New Mutations of Familial Intrahepatic Cholestasis in a Single Italian Center
Source: PLoS One. 2015 Dec 17;10(12):e0145021. doi: 10.1371/journal.pone.0145021 (PMC4683058; doi:10.1371/journal.pone.0145021)
Supplement: S1 Fig — The grayed residues in MDR1A sequence correspond to a disordered region with atomic coordinates missing in the crystal structure employed as the template for homology modeling (PDB accession code 4M1M, chain A). The two grayed regions in BSEP sequence highlight, in the order, an insertion with respect to MDR1A and to a region homologous to the disordered region of MDR1A. Amino acids highlighted in red indicate the residues affected by the mutations E135K, R1050C, R1153H, and the position of the first residue involved in the L1099LfsX38. (DOC) [file pone.0145021.s001.doc]

**Pairwise sequence alignment used for homology modeling of human BSEP.**

Pairwise sequence alignment (clustal format) of human BSEP and multidrug resistance protein 1A (MDR1A) from mouse. The grayed residues in MDR1A sequence correspond to a disordered region with atomic coordinates missing in the crystal structure employed as the template for homology modeling (PDB accession code 4M1M, chain A). The two grayed regions in BSEP sequence highlight, in the order, an insertion with respect to MDR1A and to a region homologous to the disordered region of MDR1A. Amino acids highlighted in red indicate the residues affected by the mutations E135K, R1050C, R1153H, and the position of the first residue involved in the L1099LfsX38.

ABCBB (45)VGFFQLFRFSSSTDIWLMFVGSLCAFLHGIAQPGVLLIFGTMTDVFIDYDVELQELQIPG(104)

MDR1A VSVLTMFRYAGWLDRLYMLVGTLAAIIHGVALPLMMLIFGDMTDSFASVGQVSKQST---

*..: :**::. * *:**:*.*::**:* * ::**** *** * . . ::

(E135)

ABCBB (105)KACVNNTIVWTNSSLNQNMTNGTRCGLLNIESEMIKFASYYAGIAVAVLITGYIQICFWV(164)

MDR1A ----------------QMSEADKRAMFAKLEEEMTTYAYYYTGIGAGVLIVAYIQVSFWC

* ..*. : ::*.** .:* **:**...***..***:.**

ABCBB (165)IAAARQIQKMRKFYFRRIMRMEIGWFDCNSVGELNTRFSDDINKINDAIADQMALFIQRM(224)

MDR1A LAAGRQIHKIRQKFFHAIMNQEIGWFDVHDVGELNTRLTDDVSKINEGIGDKIGMFFQAM

:**.***:*:*: :*: **. ****** :.*******::**:.***:.*.*::.:*:* *

ABCBB (225)TSTICGFLLGFFRGWKLTLVIISVSPLIGIGAATIGLSVSKFTDYELKAYAKAGVVADEV(284)

MDR1A ATFFGGFIIGFTRGWKLTLVILAISPVLGLSAGIWAKILSSFTDKELHAYAKAGAVAEEV

:: : **::** *********:::**::*:.*. . :*.*** **:******.**:**

ABCBB (285)ISSMRTVAAFGGEKREVERYEKNLVFAQRWGIRKGIVMGFFTGFVWCLIFLCYALAFWYG(344)

MDR1A LAAIRTVIAFGGQKKELERYNNNLEEAKRLGIKKAITANISMGAAFLLIYASYALAFWYG

::::*** ****:*:*:***::** *:* **:*.*. .: * .: **: .********

ABCBB (345)STLVLDEGEYTPGTLVQIFLSVIVGALNLGNASPCLEAFATGRAAATSIFETIDRKPIID(404)

MDR1A TSLVI-SKEYSIGQVLTVFFSVLIGAFSVGQASPNIEAFANARGAAYEVFKIIDNKPSID

::**: . **: * :: :*:**::**:.:*:*** :****..*.** .:*: **.** **

ABCBB (405)CMSEDGYKLDRIKGEIEFHNVTFHYPSRPEVKILNDLNMVIKPGEMTALVGPSGAGKSTA(464)

MDR1A SFSKSGHKPDNIQGNLEFKNIHFSYPSRKEVQILKGLNLKVKSGQTVALVGNSGCGKSTT

.:*:.*:* *.*:*::**:*: * **** **:**:.**: :*.*: .**** **.****:

ABCBB (465)LQLIQRFYDPCEGMVTVDGHDIRSLNIQWLRDQIGIVEQEPVLFSTTIAENIRYGREDAT(524)

MDR1A VQLMQRLYDPLDGMVSIDGQDIRTINVRYLREIIGVVSQEPVLFATTIAENIRYGREDVT

:**:**:*** :***::**:***::*:::**: **:*.******:*************.*

ABCBB (525)MEDIVQAAKEANAYNFIMDLPQQFDTLVGEGGGQMSGGQKQRVAIARALIRNPKILLLDM(584)

MDR1A MDEIEKAVKEANAYDFIMKLPHQFDTLVGERGAQLSGGQKQRIAIARALVRNPKILLLDE

*::* :*.******:***.**:******** *.*:*******:******:*********

ABCBB (585)ATSALDNESEAMVQEVLSKIQHGHTIISVAHRLSTVRAADTIIGFEHGTAVERGTHEELL(644)

MDR1A ATSALDTESEAVVQAALDKAREGRTTIVIAHRLSTVRNADVIAGFDGGVIVEQGNHDELM

******.****:** .*.* :.*:* * :******** **.* **: *. **:*.*:**:

ABCBB (645)ERKGVYFTLVTLQSQGNQ-ALNEEDIKDATEDDMLARTFSRGSYQDSLRASIRQRSKSQL(703)

MDR1A REKGIYFKLVMTQTAGNEIELGNEACKSKDEIDNLDM-----SSKDSGSSLIRRRSTRKS

..**:**.** *: **: *.:* *. * * * * :** : **:**. :

ABCBB (704)SYLVHEPPLAVVDHKSTYEEDRKDKDIPVQEEVEPAPVRRILKFSAPEWPYMLVGSVGAA(763)

MDR1A ICGPHD------------QDRKLSTKEALDEDVPPASFWRILKLNSTEWPYFVVGIFCAI

*: :: : ... .::*:* **.. ****:.:.****::** . *

ABCBB (764)VNGTVTPLYAFLFSQILGTFSIPD-KEEQRSQINGVCLLFVAMGCVSLFTQFLQGYAFAK(822)

MDR1A INGGLQPAFSVIFSKVVGVFTNGGPPETQRQNSNLFSLLFLILGIISFITFFLQGFTFGK

:** : * ::.:**:::*.*: . * **.: * ..***: :* :*::* ****::*.*

ABCBB (823)SGELLTKRLRKFGFRAMLGQDIAWFDDLRNSPGALTTRLATDASQVQGAAGSQIGMIVNS(882)

MDR1A AGEILTKRLRYMVFKSMLRQDVSWFDDPKNTTGALTTRLANDAAQVKGATGSRLAVIFQN

:**:****** : *::** **::**** :*:.********.**:**:**:**::.:*.:.

ABCBB (883)FTNVTVAMIIAFSFSWKLSLVILCFFPFLALSGATQTRMLTGFASRDKQALEMVGQITNE(942)

MDR1A IANLGTGIIISLIYGWQLTLLLLAIVPIIAIAGVVEMKMLSGQALKDKKELEGSGKIATE

::*: ..:**:: :.*:*:*::*.:.*::*::*..: :**:* * :**: ** *:*:.*

ABCBB (943)ALSNIRTVAGIGKERRFIEALETELEKPFKTAIQKANIYGFCFAFAQCIMFIANSASYRY(1002)

MDR1A AIENFRTVVSLTREQKFETMYAQSLQIPYRNAMKKAHVFGITFSFTQAMMYFSYAAAFRF

*:.*:***..: :*::* .*: *::.*::**:::*: *:*:*.:*::: :*::*:

(R1050)

ABCBB (1003)GGYLISNEGLHFSYVFRVISAVVLSATALGRAFSYTPSYAKAKISAARFFQLLDRQPPIS(1062)

MDR1A GAYLVTQQLMTFENVLLVFSAIVFGAMAVGQVSSFAPDYAKATVSASHIIRIIEKTPEID

*.**:::: : *. *: *:**:*:.* *:*:. *::*.****.:**::::::::: * *.

(L1099LfsX38)

ABCBB (1063)VYNTAGEKWDNFQGKIDFVDCKFTYPSRPDSQVLNGLSVSISPGQTLAFVGSSGCGKSTS(1122)

MDR1A SYSTQGLKPNMLEGNVQFSGVVFNYPTRPSIPVLQGLSLEVKKGQTLALVGSSGCGKSTV

*.* * * : ::*:::* . *.**:**. **:***:.:. *****:**********

(R1153)

ABCBB (1123)IQLLERFYDPDQGKVMIDGHDSKKVNVQFLRSNIGIVSQEPVLFACSIMDNIKYGDNTKE(1182)

MDR1A VQLLERFYDPMAGSVFLDGKEIKQLNVQWLRAQLGIVSQEPILFDCSIAENIAYGDNSRV

:********* *.*::**:: *::***:**:::*******:** *** :** ****::

ABCBB (1183)IPMERVIAAAKQAQLHDFVMSLPEKYETNVGSQGSQLSRGEKQRIAIARAIVRDPKILLL(1242)

MDR1A VSYEEIVRAAKEANIHQFIDSLPDKYNTRVGDKGTQLSGGQKQRIAIARALVRQPHILLL

:. *.:: ***:*::*:*: ***:**:*.**.:*:*** *:*********:**:*:****

ABCBB (1243)DEATSALDTESEKTVQVALDKAREGRTCIVIAHRLSTIQNADIIAVMAQGVVIEKGTHEE(1302)

MDR1A DEATSALDTESEKVVQEALDKAREGRTCIVIAHRLSTIQNADLIVVIQNGKVKEHGTHQQ

*************.** *************************:*.*: :* * *:***::

ABCBB (1303)LMAQKGAYYKLVTTGS(1318)

MDR1A LLAQKGIYFSMVSVQA

*:**** *:.:*:. :
