# Supplementary material for: Genetics and Molecular Modeling of New Mutations of Familial Intrahepatic Cholestasis in a Single Italian Center
Source: PLoS One. 2015 Dec 17;10(12):e0145021. doi: 10.1371/journal.pone.0145021 (PMC4683058; doi:10.1371/journal.pone.0145021)
Supplement: S2 Fig — The grayed residues in MDR1A sequence correspond to a disordered region with atomic coordinates missing in the crystal structure employed as the template for homology modeling (PDB entry 3G5U, chain A). (DOC) [file pone.0145021.s002.doc]

**Pairwise sequence alignment employed for homology modeling of human ABCB4.**

Pairwise sequence alignment of human ABCB4 and multidrug resistance protein 1A (MDR1A) from mouse. The grayed residues in MDR1A sequence correspond to a disordered region with atomic coordinates missing in the crystal structure employed as the template for homology modeling (PDB entry 3G5U, chain A).

MDR3_HUMAN IGVLTLFRYSDWQDKLFMSLGTIMAIAHGSGLPLMMIVFGEMTDKFVDTAGNFSFPVNFS

MDR1A_MOUSE VSVLTMFRYAGWLDRLYMLVGTLAAIIHGVALPLMMLIFGDMTDSFASVGNVSKNSTNMS

:.***:***:.* *:*:* :**: ** ** .*****::**:***.*..... . ..*:*

MDR3_HUMAN L-SLLNPGKILEEEMTRYAYYYSGLGAGVLVAAYIQVSFWTLAAGRQIRKIRQKFFHAIL

MDR1A_MOUSE EADKRAMFAKLEEEMTTYAYYYTGIGAGVLIVAYIQVSFWCLAAGRQIHKIRQKFFHAIM

. ****** *****:*:*****:.******** *******:**********:

MDR3_HUMAN RQEIGWFDINDTTELNTRLTDDISKISEGIGDKVGMFFQAVATFFAGFIVGFIRGWKLTL

MDR1A_MOUSE NQEIGWFDVHDVGELNTRLTDDVSKINEGIGDKIGMFFQAMATFFGGFIIGFTRGWKLTL

.*******::*. *********:***.******:******:****.***:** *******

MDR3_HUMAN VIMAISPILGLSAAVWAKILSAFSDKELAAYAKAGAVAEEALGAIRTVIAFGGQNKELER

MDR1A_MOUSE VILAISPVLGLSAGIWAKILSSFTDKELHAYAKAGAVAEEVLAAIRTVIAFGGQKKELER

**:****:*****.:******:*:**** ***********.*.***********:*****

MDR3_HUMAN YQKHLENAKEIGIKKAISANISMGIAFLLIYASYALAFWYGSTLVISKEYTIGNAMTVFF

MDR1A_MOUSE YNNNLEEAKRLGIKKAITANISMGAAFLLIYASYALAFWYGTSLVISKEYSIGQVLTVFF

*:::**:**.:******:****** ****************::*******:**:.:****

MDR3_HUMAN SILIGAFSVGQAAPCIDAFANARGAAYVIFDIIDNNPKIDSFSERGHKPDSIKGNLEFND

MDR1A_MOUSE SVLIGAFSVGQASPNIEAFANARGAAYEVFKIIDNKPSIDSFSKSGHKPDNIQGNLEFKN

*:**********:* *:********** :*.****:*.*****: *****.*:*****::

MDR3_HUMAN VHFSYPSRANVKILKGLNLKVQSGQTVALVGSSGCGKSTTVQLIQRLYDPDEGTINIDGQ

MDR1A_MOUSE IHFSYPSRKEVQILKGLNLKVKSGQTVALVGNSGCGKSTTVQLMQRLYDPLDGMVSIDGQ

:******* :*:*********:*********.***********:****** :* :.****

MDR3_HUMAN DIRNFNVNYLREIIGVVSQEPVLFSTTIAENICYGRGNVTMDEIKKAVKEANAYEFIMKL

MDR1A_MOUSE DIRTINVRYLREIIGVVSQEPVLFATTIAENIRYGREDVTMDEIEKAVKEANAYDFIMKL

***.:**.****************:******* *** :******:*********:*****

MDR3_HUMAN PQKFDTLVGERGAQLSGGQKQRIAIARALVRNPKILLLDEATSALDTESEAEVQAALDKA

MDR1A_MOUSE PHQFDTLVGERGAQLSGGQKQRIAIARALVRNPKILLLDEATSALDTESEAVVQAALDKA

*::************************************************ ********

MDR3_HUMAN REGRTTIVIAHRLSTVRNADVIAGFEDGVIVEQGSHSELMKKEGVYFKLVNMQTSGSQIQ

MDR1A_MOUSE REGRTTIVIAHRLSTVRNADVIAGFDGGVIVEQGNHDELMREKGIYFKLVMTQTAGNEIE

*************************:.*******.*.***:::*:***** **:*.:*:

MDR3_HUMAN --SEEFELNDEKAATRMAPNGWKSRLFRHSTQKNLKNSQMCQKSLDVETDGLEANVPPVS

MDR1A_MOUSE LGNEACKSKDEIDNLDMSSKDSGSSLIRRRSTRKSICGPHDQDRKLSTKEALDEDVPPAS

.* : :** *:.:. * *:*: : :: . *. .:.*: :***.*

MDR3_HUMAN FLKVLKLNKTEWPYFVVGTVCAIANGGLQPAFSVIFSEIIAIFGPGDDAVK-QQKCNIFS

MDR1A_MOUSE FWRILKLNSTEWPYFVVGIFCAIINGGLQPAFSVIFSKVVGVFTNGGPPETQRQNSNLFS

* ::****.********* .*** *************:::.:* *. . . :*:.*:**

MDR3_HUMAN LIFLFLGIISFFTFFLQGFTFGKAGEILTRRLRSMAFKAMLRQDMSWFDDHKNSTGALST

MDR1A_MOUSE LLFLILGIISFITFFLQGFTFGKAGEILTKRLRYMVFKSMLRQDVSWFDDPKNTTGALTT

*:**:******:*****************:*** *.**:*****:***** **:****:*

MDR3_HUMAN RLATDAAQVQGATGTRLALIAQNIANLGTGIIISFIYGWQLTLLLLAVVPIIAVSGIVEM

MDR1A_MOUSE RLANDAAQVKGATGSRLAVIFQNIANLGTGIIISLIYGWQLTLLLLAIVPIIAIAGVVEM

***.*****:****:***:* *************:************:*****::*:***

MDR3_HUMAN KLLAGNAKRDKKELEAAGKIATEAIENIRTVVSLTQERKFESMYVEKLYGPYRNSVQKAH

MDR1A_MOUSE KMLSGQALKDKKELEGSGKIATEAIENFRTVVSLTREQKFETMYAQSLQIPYRNAMKKAH

*:*:*:* :******.:**********:*******:*:***:**.:.* ****:::***

MDR3_HUMAN IYGITFSISQAFMYFSYAGCFRFGAYLIVNGHMRFRDVILVFSAIVFGAVALGHASSFAP

MDR1A_MOUSE VFGITFSFTQAMMYFSYAACFRFGAYLVTQQLMTFENVLLVFSAIVFGAMAVGQVSSFAP

::*****::**:******.********:.: * *.:*:**********:*:*:.*****

MDR3_HUMAN DYAKAKLSAAHLFMLFERQPLIDSYSEEGLKPDKFEGNITFNEVVFNYPTRANVPVLQGL

MDR1A_MOUSE DYAKATVSASHIIRIIEKTPEIDSYSTQGLKPNMLEGNVQFSGVVFNYPTRPSIPVLQGL

*****.:**:*:: ::*: * ***** :****: :***: *. ********..:******

MDR3_HUMAN SLEVKKGQTLALVGSSGCGKSTVVQLLERFYDPLAGTVFVDFGFQLLDGQEAKKLNVQWL

MDR1A_MOUSE SLEVKKGQTLALVGSSGCGKSTVVQLLERFYDPMAGSV-------FLDGKEIKQLNVQWL

*********************************:**:* :***:* *:******

MDR3_HUMAN RAQLGIVSQEPILFDCSIAENIAYGDNSRVVSQDEIVSAAKAANIHPFIETLPHKYETRV

MDR1A_MOUSE RAQLGIVSQEPILFDCSIAENIAYGDNSRVVSYEEIVRAAKEANIHQFIDSLPDKYNTRV

******************************** :*** *** **** **::**.**:***

MDR3_HUMAN GDKGTQLSGGQKQRIAIARALIRQPQILLLDEATSALDTESEKVVQEALDKAREGRTCIV

MDR1A_MOUSE GDKGTQLSGGQKQRIAIARALVRQPHILLLDEATSALDTESEKVVQEALDKAREGRTCIV

*********************:***:**********************************

MDR3_HUMAN IAHRLSTIQNADLIVVFQNGRVKEHGTHQQLLAQKGIYFSMVSVQA

MDR1A_MOUSE IAHRLSTIQNADLIVVIQNGKVKEHGTHQQLLAQKGIYFSMVSVQA

****************:***:*************************
