# Supplementary material for: Genetics and Molecular Modeling of New Mutations of Familial Intrahepatic Cholestasis in a Single Italian Center
Source: PLoS One. 2015 Dec 17;10(12):e0145021. doi: 10.1371/journal.pone.0145021 (PMC4683058; doi:10.1371/journal.pone.0145021)
Supplement: S1 Table — (DOCX) [file pone.0145021.s004.docx]

| **Table 1 Sup.Material: Primers for PCR and qPCR amplification of *ATP8B1*, *ABCB11* and *ABCB4* genomic DNA** | |
| --- | --- |
| FIC1-QPCR-ex1UTR-F | CAGCGAACGCCAGgtaag |
| FIC1-QPCR-ex1UTR-R | aggggcttgggaaggtct |
| FIC1-QPCR-ex1F | TGACGAAGTGGTTCCCTACA |
| FIC1-QPCR-ex1R | CCCTGTTGACTCGGTTTTGT |
| FIC1-QPCR-ex2F | ACATGGCAAGTCAAAGCAAA |
| FIC1-QPCR-ex2R | AAAGTGAGGTTGTTCGTGGT |
| FIC1-QPCR-ex18F | CCTATGCCTTTGCTACAAGGA |
| FIC1-QPCR-ex18R | CACACTGGCAGCCATAAACTT |
| FIC1-EX1F | TGCAGGCAGTATTCAACCAA |
| FIC1-EX1R | CACGCAAAATAGACCGATCA |
| FIC1-EX2F | GGGAAGACACAATTCAACTCA |
| FIC-1-EX2R | TGCATTTTAATGAAAACAATTCAGA |
| FIC1-EX3F | GGGACTTGTGACTTTTGCAG |
| FIC1-EX3R | TGTGTCTACAGCTTAAATGTTATCGAG |
| FIC1-EX4/5F | ATGCCTTCATGGACACACAC |
| FIC1-EX4/5R | TGCATTACATCCTTGAAACTCA |
| FIC1-EX6/7F | TCCCTTGCCTGTAACTTAAAATG |
| FIC1-EX6/7R | GGAATGAATGTGCCTTCAAA |
| FIC1-EX8/9F | GGCTTCATGTCCAGGTATGG |
| FIC1-EX8/9R | CATAGCTGATTAATTTCCCAAGAA |
| FIC1-EX10F | TGAATTGATTTTTCCCAACTGA |
| FIC1-EX10R | CGATAATATTAGTGCAAAAGACAGC |
| FIC1-EX11F | GGAAATGCAAGAGGTTGGAA |
| FIC1-EX11R | GGCACTATGTTGGGAGAAGG |
| FIC1-EX12F | TCCGAGCTCTCTACGGAAAA |
| FIC1-EX12R | TAGCAGCAGGACTCTGCATC |
| FIC1-EX13F | AAGCAAAGCCAGGTAAGGAG |
| FIC1-EX13R | CAGCATCCCAAACGATTCTT |
| FIC1-EX14F | TGAAACCTTGCCTTTGAAGAA |
| FIC1-EX14R | TGCATTTGAGCCATAAGCAG |
| FIC1-EX15F | GGCCTCAACAATGAGCTCTG |
| FIC1-EX15R | TGGGCACAAGCAACATCTAA |
| FIC1-EX16F | AGCCTGGACAACAGAGCAAG |
| FIC1-EX16R | TCAGAATCCCTTGCAGAAAGA |
| FIC1-EX17F | ATTCTTTGCATTGGTGGATTT |
| FIC1-EX17R | TTCTGCTTAGAATATCAAAGAAAAAGT |
| FIC1-EX18F | CAGAAAACTTTTGGTTTGTTTGC |
| FIC1-EX18R | CGGCAGCTATAAATATTTGTGAATTA |
| FIC1-EX19F | CCTAGGCAGTGGGAGTGAGA |
| FIC1-EX19R | TTGCATTTGCAAAGATGAGC |
| FIC1-EX20F | TCTCAGAGTCAAGGGCCTATTT |
| FIC1-EX20R | GCATCTAAAAGTGGCTCCAAA |
| FIC1-EX21F | TCTTGGGAATGGTACTCCTG |
| FIC1-EX21R | CCCTACACATTCCAGCCATT |
| FIC1-EX22F | CAACTGATGTTTTGCCTCACA |
| FIC1-EX22R | GACACAGCCCCAAAACTACAA |
| FIC1-EX23F | GGCATGTGTCTTTGTTGGTG |
| FIC1-EX23R | CGTTTGCTTGGGACTTCTCT |
| FIC1-EX24F | TGACTGCTTTGACCTGATGAA |
| FIC1-EX24R | AGTGGGAGTCAGGTGGATGT |
| FIC1-EX25F | AACTCCCCAAAATGAGTGCT |
| FIC1-EX25R | GACATTTGTCTGTACATTTATTTTTGG |
| FIC1-EX26F | CACCACACCTGGCGAAATA |
| FIC1-EX26R | ACGCTTTGGTTTCTGTGAGG |
| FIC1-EX27F | CCTCCCTGGTGTGGATCTTA |
| FIC1-EX27R | AAAAATAGACGTGCTTTGTGG |
| FIC2-EX1F | TGACTGTGGCTTATCTTTCCTG |
| FIC2-EX1R | TGCTCCTTGAAACTTGACCA |
| FIC2-EX2F | CTGCGTTGCATTTTGTCATT |
| FIC2-EX2R | TCTCTGCTTTGTGCCTTTGA |
| FIC2-EX3F | AAAATCACCACCTAGGGAGAA |
| FIC2-EX3R | GATTTAACACTCCCCTCATGATCT |
| FIC2-EX4F | TCCTCCTACCTCTCCTGCTCT |
| FIC2-EX4R | TCAGCCAGTAAAATCCCCTCT |
| FIC2-EX5F | GGTGGCTTGATCCTACTTGC |
| FIC2-EX5R | GTGGCAACACATTGCATCTC |
| FIC2-EX6F | TTTCCCCCTTTTCTCAACTG |
| FIC2-EX6R | GCCACATATGAAAGCCCAAT |
| FIC2-EX7F | TCCAAGGGTGATAGGGATAGA |
| FIC2-EX7R | TCAGGAAAAGGGACTCAAGC |
| FIC2-EX8F | CCTAATTTCTTGGACTTCACATTT |
| FIC2-EX8R | CGCTTTGCACAAACTGAGAG |
| FIC2-EX9F | CATTTTGTAAAACCACTGCATCA |
| FIC2-EX9R | CAGAAGGAAATGCTATGTCTCG |
| FIC2-EX10F | TCTGCGTTAACATGGAAGACC |
| FIC2-EX10R | CAGCCCCCACCTGTTAATG |
| FIC2-EX11F | CGCCAAAGATGTTTATTTGAA |
| FIC2-EX11R | CAGGCTTCAGAAAATGAGCA |
| FIC2-EX12F | CACAAAGCATCTGCACCTGT |
| FIC2-EX12R | GCCATTTGCACTTTACTGTCC |
| FIC2-EX13F | CCAAAAGTTGTGATGTTGTGC |
| FIC2-EX13R | CAGGCATGAAACTAAAACATGG |
| FIC2-EX14F | TCACTGTCAGAAGCCATCAAA |
| FIC2-EX14R | ACAAGGAGCTGCCTTTCCTG |
| FIC2-EX15F | TGATGCAAAGGTCAGTGTCAG |
| FIC2-EX15R | TGAAATACATAGAAAACCGTAAAGCA |
| FIC2-EX16F | AAAGAATTCTACTTGGATATGGTTCTG |
| FIC2-EX16R | TCTGAGGATTAGGACTACAGAGGA |
| FIC2-EX17F | CCAGTTGATCCTGCTCCAAT |
| FIC2-EX17R | CAACAGTCCCCAGGAGAGAC |
| FIC2-EX18F | TGTGAATGCCAAAGGATCTG |
| FIC2-EX18R | TGAAAACAAAGAGCGGACTTATC |
| FIC2-EX19/20F | TTTGGACAGATATATAATGACATGG |
| FIC2-EX19/20R | GCAGGTGATTGTCAGAATGC |
| FIC2-EX21F | AAAAGCGACTGTGTGTCTGA |
| FIC2-EX21R | TGATAGCCACTCAGCCATGA |
| FIC2-EX22F | GCCACTGAAATGTCACGAAA |
| FIC2-EX22R | CAGAACCAGGCTATTCCTTCC |
| FIC2-EX23F | GGGCTAGATCCCCAAACTTC |
| FIC2-EX23R | TTTCCAGCTTCATCCCTGTC |
| FIC2-EX24F | GAAGGTATCTCAAGCAGGGATTT |
| FIC2-EX24R | AAGCCCACTTTTAGGGGTTG |
| FIC2-EX25F | CCAAATGTCCTGCATAACACC |
| FIC2-EX25R | CTCCCCATCCTTGTCTCTCA |
| FIC2-EX26F | TTCAGTACAGCACAGGAGCAA |
| FIC2-EX26R | AAGGACAAATTTTACAGCAAAAGA |
| FIC2-EX27F | GGATTGTTATTCAGGTCGTGTT |
| FIC2-EX27R | AACTGGTGCGTCATGTGTGT |
| MDR3-EX2F | CGGAGAGGGTGTACTTGGTT |
| MDR3-EX2R | AACACGTTTGCTCCAAGGTC |
| MDR3-EX3F | AATTTTGACATTTTGTGAACATCA |
| MDR3-EX3R | CTCCCAAAGTGCTGGGATTA |
| MDR3-EX4F | GGAGAAATTCCATTCCACAGC |
| MDR3-EX4R | TCAACTCCCAAATTTTTACCC |
| MDR3-EX5F | TTAAGTGTGCAAAGAAAAGTGTTT |
| MDR3-EX5R | GGATTTGGGAGCAAAAATGA |
| MDR3-EX6F | AGCCTGGGTGACAGAGTGAG |
| MDR3-EX6R | TCTTTCCTTGACATATTTTCACACA |
| MDR3-EX7F | AAACCTCCTGCCTGTAACCA |
| MDR3-EX7R | CACATAAAAAGGCCCAGCTT |
| MDR3-EX8F | AGGAGAGGGTTTGGGAAGAA |
| MDR3-EX8R | CCACAAAGAAGAAGCAACAAAA |
| MDR3-EX9F | TGTGACTCGGACTATGGATTG |
| MDR3-EX9R | GCGATATCAAAGAAAAGAGAAGG |
| MDR3-EX10F | TGAATGCCAGAATGTGACTTAAA |
| MDR3-EX10R | GCCAGATTTAATTATACAAGCTCAAA |
| MDR3-EX11/12F | CAACTTGTTTGTGCTATGATGGA |
| MDR3-EX11/12R | ACCAATTTCAAAGGGCCAAT |
| MDR3-EX13F | TGAATGGTCCTGATACTTCAGC |
| MDR3-EX13R | AAACTCAGTCCTATGAGGTGAAA |
| MDR3-EX14F | CAAAGCTCCATGTTGTCTTTATG |
| MDR3-EX14R | TGTTTCTCAGCCCAGACTCC |
| MDR3-EX15F | CCTGATGCACCACATTTTTG |
| MDR3-EX15R | TGCTCAGTATAGCATTCACTGGA |
| MDR3-EX16F | CACACACACACACACACACACT |
| MDR3-EX16R | CAGTCATCTGTGCCTGAAAAA |
| MDR3-EX17F | ATGGCCATGCCTTTTCTATG |
| MDR3-EX17R | AAGCAGCAGCTGATGAATTG |
| MDR3-EX18F | TGAGAGGAATGAAGAAGGATCA |
| MDR3-EX18R | ACTTGATGAGAAAGGCAAATCA |
| MDR3-EX19/20F | AAAGATCAAAAGAGACCATAGCC |
| MDR3-EX19/20R | AAGTGTGGGTATGCTACATGCT |
| MDR3-EX21F | CATGCATTTGGAGCTTAAAACTA |
| MDR3-EX21R | AAACAACACTTAACACCAATTGAAA |
| MDR3-EX22F | TGAAAACACCACTTCTAATGAAGG |
| MDR3-EX22R | TTTGGGACAATAATTCAGCCTTA |
| MDR3-EX23F | AAGCCGTGCTCTTTCCACTA |
| MDR3-EX23R | CCCTGACCTCATCTTTGGAC |
| MDR3-EX24F | GGGGAGAAAGGGGATGATTA |
| MDR3-EX24R | CAAGCATCATCAGGCATCAG |
| MDR3-EX25F | CAGTCTTTGGTAAAGTTTCCTTGAA |
| MDR3-EX25R | ATTGGTTGGGCCAATTAAAA |
| MDR3-EX26F | AACGATCCTCCAAATGGACA |
| MDR3-EX26R | TGGTAATTGTTTGGGGGATAA |
| MDR3-EX27F | AAACACTCTGTTAAGTTGAAACAACG |
| MDR3-EX27R | CATGGTTGACAGCAAAATCC |
| MDR3-EX28F | TTGGGATAAGGTGTCTGTCTGA |
| MDR3-EX28R | TGTTTTATGATGACAAACCAGAAA |
